# Results from a comparative study to evaluate the treatment effectiveness of a nonpneumatic compression device vs an advanced pneumatic compression device for lower extremity lymphedema swelling (TEAYS study)

**DOI:** 10.1016/j.jvsv.2024.101965

**Published:** 2024-09-01

**Authors:** Michael Barfield, Ron Winokur, Todd Berland, Sandi Davis, Vicky Ralph, Nancy Chatham, Stanley Rockson, Thomas S. Maldonado

**Affiliations:** a University Surgical Associates, University of Tennessee Health Science Center, Nashville, TN; b Vascular and Interventional Radiology, Weill Cornell Medical Center, New York, NY; c Vascular Surgery, New York University, New York, NY; d Davis Care Physical Therapy, New York, NY; e Physical Medicine and Rehabilitation, University of Colorado, Denver, CO; f St. Johns Regional Wound Clinic, Hospital Sisters Health Center, Springfield, IL; g Department of Cardiovascular Medicine, Stanford University, Stanford, CA

**Keywords:** NPCD, Nonpneumatic compression, Pneumatic compression, Dayspring, Lymphedema treatment, Lower extremity lymphedema, Phlebolymphedema

## Abstract

**Objective:**

Advanced pneumatic compression devices (APCDs) have been shown to be effective in treatment of lower extremity lymphedema in the home setting. However, adherence to self-care has been poor, and APCDs require patients to remain immobile during treatment. We evaluated the safety and efficacy of a novel nonpneumatic compression device (NPCD) for treating lower extremity lymphedema vs an APCD.

**Methods:**

A randomized, crossover head-to-head study was performed at nine sites in 2023. Patients were randomized to either the NPCD or a commercially available APCD. Patients used the randomly assigned initial device for 90 days with a 4-week washout period before a comparable 90-day use of the second device.

**Results:**

A total of 71 patients (108 affected limbs) with lower extremity lymphedema were analyzed. Compared with the APCD, the NPCD was associated with a greater mean decrease in limb edema volume (a mean limb volume decrease of 369.9 ± 68.19 mL [*P* < .05] vs 83.1 ± 67.99 mL [*P* < .05]). Significant improvement in Quality of Life was achieved for NPCD and but not for APCD treatment (score improvement of 1.01 ± 0.23 [*P* < .05] for NPCD vs 0.17 ± 0.18 [*P* > .05] for APCD). Patients reported greater adherence (81% vs 56%; *P* < .001) and satisfaction with the NPCD (78% vs 22%) compared with APCD. No device-related adverse events were reported.

**Conclusions:**

The novel NPCD is an effective treatment for decreasing limb volume in patients with lower extremity lymphedema. The NPCD was more effective than an APCD and resulted in superior limb volume decrease, greater improved quality of life, adherence, mobility, and patient satisfaction.

**Article Highlights**

• **Type of Research:** Multicenter, prospective, randomized cross-over trial
• **Key Findings:** Data from 71 patients (108 affected limbs) with lower extremity lymphedema demonstrated significant greater improvements in limb edema and in quality of life with the use of a novel nonpneumatic compression device than with a commercially available advanced pneumatic compression device. Patients were also significantly more adherent to self-care treatment (81% vs 56%; *P* < .001), more active (91% vs 0%), and more satisfied (78% vs 22%) with the novel device.
• **Take Home Message:** The results have shown that the novel nonpneumatic compression device is safe and effective for decreasing limb volume in those with lower extremity venous/lymphatic edema. The nonpneumatic compression device is more effective and results in greater adherence and improved quality of life than a commercially available advanced pneumatic compression device.

Lymphedema is a common but often unrecognized clinical condition that is chronic and progressive in nature. The disease arises from an impaired lymphatic drainage causing an excess accumulation of interstitial fluid that results in tissue swelling and can lead to tissue and skin changes.^1^ Lymphedema impacts 250 million people worldwide, with tens of millions in the United States,^2^ and is classified either as primary or secondary. Primary lymphedema is associated with malformation of the lymphatic system, which can develop early or onset late in life, whereas secondary lymphedema is typically acquired owing to injury or insult to the lymphatic systems, such as from cancer or cancer-related treatments, injury, trauma, or venous insufficiency. In the United States, secondary lower extremity lymphedema owing to chronic venous insufficiency also known as phlebolymphedema is a leading cause.^3^

Chronic venous insufficiency has been estimated to affect as much as 50% of the adult population,^4^ and accounts for 2% of Western health care budgets.^5^ Patients with chronic venous insufficiency with venous hypertension have an increased hydrostatic pressure in capillaries and increased capillary permeability, and extravasation of proteins into intracellular spaces.^6^ The accumulation of proteins and macromolecules in interstitial spaces occurs more rapidly than lymphatic vessels can drain them, leading to the clinical development of lymphedema.^7^ In effect, the functioning of the lymphatic system is inextricably linked to the functional status of the venous system.

Unfortunately, lymphedema is a disease without a cure and has many consequences for patients, including significant impairments to their life and function.^8^ If left untreated, lymphedema can lead to severe pain, infection, ulceration, and life-threatening infections. The debilitating aspects of lymphedema include loss of mobility, impaired range of motion, skin breakdown (aside from ulceration), skin weeping, psychosocial aspects related to image, and inability to complete activities of daily living independently.^9^, ^10^, ^11^, ^12^, ^13^, ^14^ The chronic ailment and progression can magnify risk of infection, cellulitis, ulceration, as well as an increased risk of hospitalization.^9^, ^10^, ^11^, ^12^, ^13^, ^14^ Treatment goals for lymphedema include limb volume decrease and prevention of infections. Complete decongestive therapy remains the cornerstone for treatment of lymphedema. Complete decongestive therapy involves a combination treatment protocol and requires a synergy of five components that include skin care, manual lymphatic drainage, a multilayered bandaging system application with adjunct compression garments and devices, decongestive exercises, and patient education for risk decrease strategies. In the home setting, intermittent pneumatic compression devices (PCDs), including advanced pneumatic PCDs or APCDs (calibrated gradient air compressor with multiple cells), have been used in the ongoing management of this condition. Pneumatic compression treatment typically involves the patient lying down in a supine position for the entirety of the treatment duration, which can be ≤1 hour each day. Although effective, adherence remains a challenge for such therapies.^15^ Additionally, the lack of mobility during treatment remains counterproductive to recommended modalities where exercise and movement are needed for enabling the venous return and lymphatic transport and clearance.^16^^,^^17^

Novel nonpneumatic PCDs (NPCDs) for treating lower extremity lymphedema was cleared by the US Food and Drug Administration and became commercially available in the United States in 2022. Dayspring from Koya Medical (Oakland, CA) is a NPCD system consisting of a programmable controller and a limb-specific garment. It is designed to offer a distinct, multimodal treatment approach that differs from existing pneumatic compression. Specifically, the NPCD approach uses static compression, gradient sequential compression, and supports the contractions of joints and muscles, enabling patients to ambulate and activate their calf pump during treatment.^18^^,^^19^ The NPCD technology uses shape memory alloy (nickel/titanium) actuators in its garment, which contract and relax to achieve sequential gradient compression in a distal to proximal manner when specified and energized by the controller. In use, the NPCD controller is battery powered and is designed to allow the patient to retain mobility while performing their activities of daily living vs immobilizing the patient in a supine position during a pneumatic compression treatment. For the treatment of upper extremity lymphedema, clinical studies including a multicenter, randomized comparative effectiveness trial for Dayspring has demonstrated NPCD's superior usefulness in treatment effectiveness as well as improvement in quality of life (QoL) compared with pneumatic compression.^20^, ^21^, ^22^ For the treatment of lower extremity lymphedema, an open-label study demonstrated that patients achieved meaningful improvements in limb volume decrease and QoL. In this article, study results are presented from the TEAYS study (Treatment Effectiveness of a Non-Pneumatic Compression Device versus an Advanced Pneumatic Compression Device for Lower Extremity Lymphedema Swelling) comparing the treatment effectiveness between NPCD (Dayspring) and APCDs for lower extremity lymphedema.

## Methods

### Study design and eligibility

The TEAYS study was a prospective, multicenter, randomized, single, crossover clinical trial conducted across nine study sites in the United States. The study was approved by the Western institutional review board-Copernicus Group and followed a single protocol performed per good clinical practices. Eligible patients ≥18 years of age with a confirmed diagnosis of primary or secondary unilateral or bilateral lower extremity lymphedema or phlebolymphedema and willing to consent and follow the study protocol were included. The main exclusion criteria included any systemic disorder that might contraindicate the use of sequential compression, including the presence of active cellulitis and open or partially healed wounds. Additional exclusions were patients with any diagnoses of cognitive or physical impairment that would interfere with use of the device, lipedema, active or recurrent cancer (<3 months since completion of chemotherapy, radiation therapy or primary surgery for the cancer), acute infection (in the last 4 weeks), acute thrombophlebitis (in last 6 months), pulmonary embolism or deep vein thrombosis within the previous 6 months, pulmonary edema, congestive heart failure (uncontrolled or uncompensated), chronic kidney disease with acute renal failure, epilepsy, poorly controlled asthma, a condition where increased venous and lymphatic return is undesirable, women who are pregnant, women planning a pregnancy or nursing at study entry, and participation in any clinical trial of an investigational substance or device during the past 30 days.

### End points

Primary efficacy outcomes assessed in this study included change in affected limb volume between baseline (day 0) and end of treatment (day 90), change in Lymphedema Quality of Life Questionnaire (LYMQOL),^23^ and treatment adherence. Calculation of limb volume by circumference measure was performed by a trained therapist using a calibrated tape measure. Measurements were taken every 4 cm, and the volume of a truncated cone is calculated according to the Kuhnke formula, summing the eight neighboring circumference measures. Measurements were performed for all affected limbs, regardless of whether lymphedema was unilateral or bilateral.

For the QoL assessment, the LYMQOL survey was used (Appendix). The LYMQOL is a 20-item clinically validated disease-specific survey tool, that was administered at days 0 and 90 for each device treatment period. The survey assesses the effects of lymphedema on QOL through both an overall score (scored 1-10) and four subscores: symptoms (pain, swelling, numbness), body image and appearance, function (activities of daily living; eg, eating, writing, and dressing), and mood (ie, sleep disruption, depression, and irritability). The subdomains are scored from 1 (not at all) to 4 (a lot). The total score is calculated by summing all scores and dividing by the total number of items. The domain-specific subscores reflect improvement as a lower score, and the overall QOL score reflects improvement by a higher score. Changes from days 0 to day 90 for the total score and each subscore were calculated.

Treatment adherence was reported through patient diaries over the 90-day course of treatment for each device. Adherence was calculated as the percentage of reported daily use (minimum of 1 hour) over the treatment period (ie, patients who used device for the entire 90 days achieved 100% adherence, whereas those who used device every other day reported 50% adherence).

Secondary outcomes included safety as measured by device-related adverse events (AEs) (eg, pressure-induced wounds, allergic reactions to garments, pain from use of device, or burns) throughout the course of study, and a patient survey administered at the end of the study. The survey evaluated the patient's preference for treatment modality as well as their perceived mobility and device portability during treatment and whether they experienced decreased use of their compression garments during each treatment period. Reports on truncal swelling before and after device use were also collected.

Additional disease-related health episodes and resource use information were collected including episodes of cellulitis, ulceration, hospitalization, lymphedema-related physical therapy visits, and compression stocking use over the past 12 months before device use and during the study duration with each device treatment.

### Randomization and treatment

**Fig 1:**
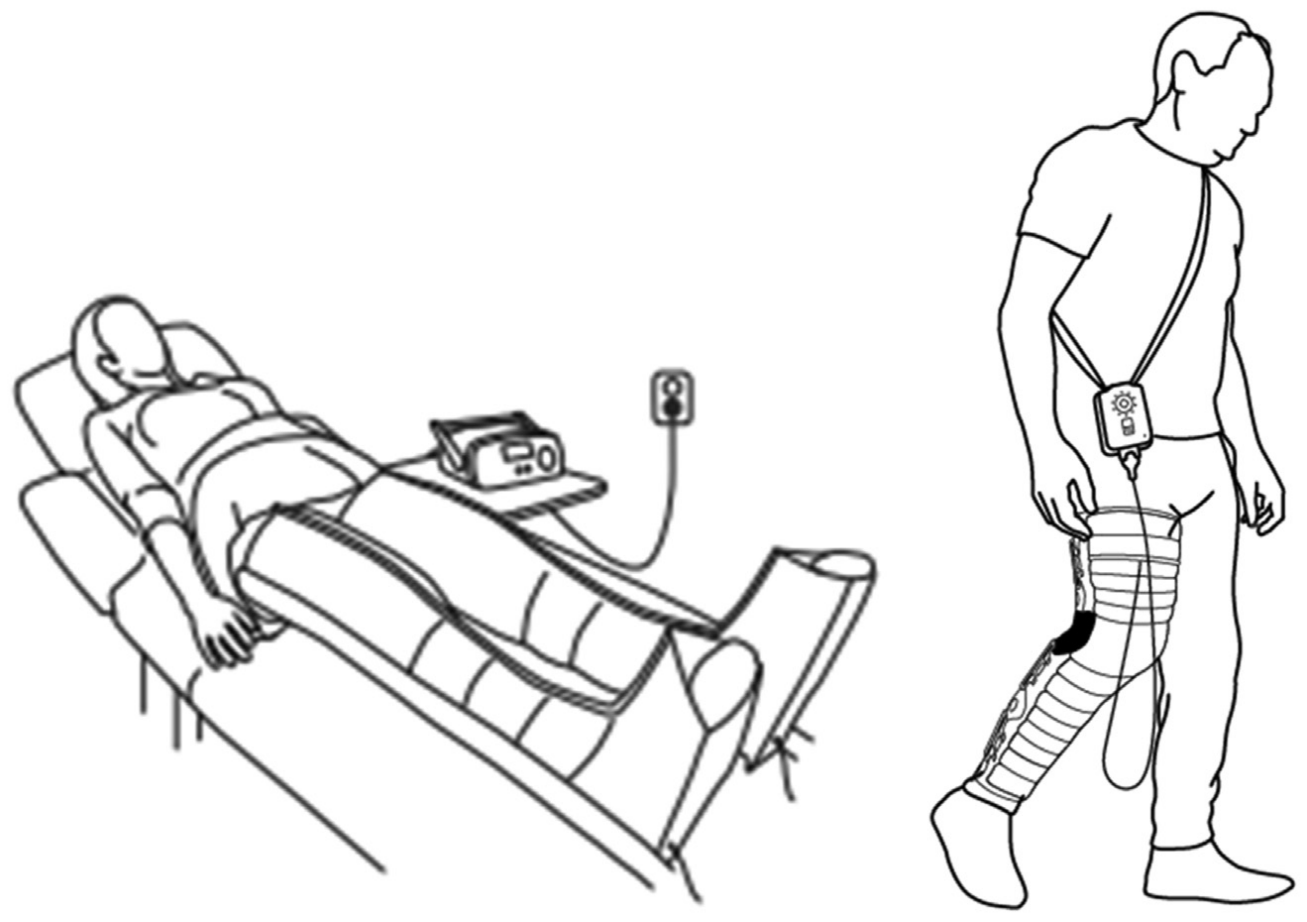
An outline of the advanced pneumatic compression device (APCD) device (supine) and outline of the nonpneumatic compression device (NPCD) device (Dayspring, in motion).

**Fig 2:**
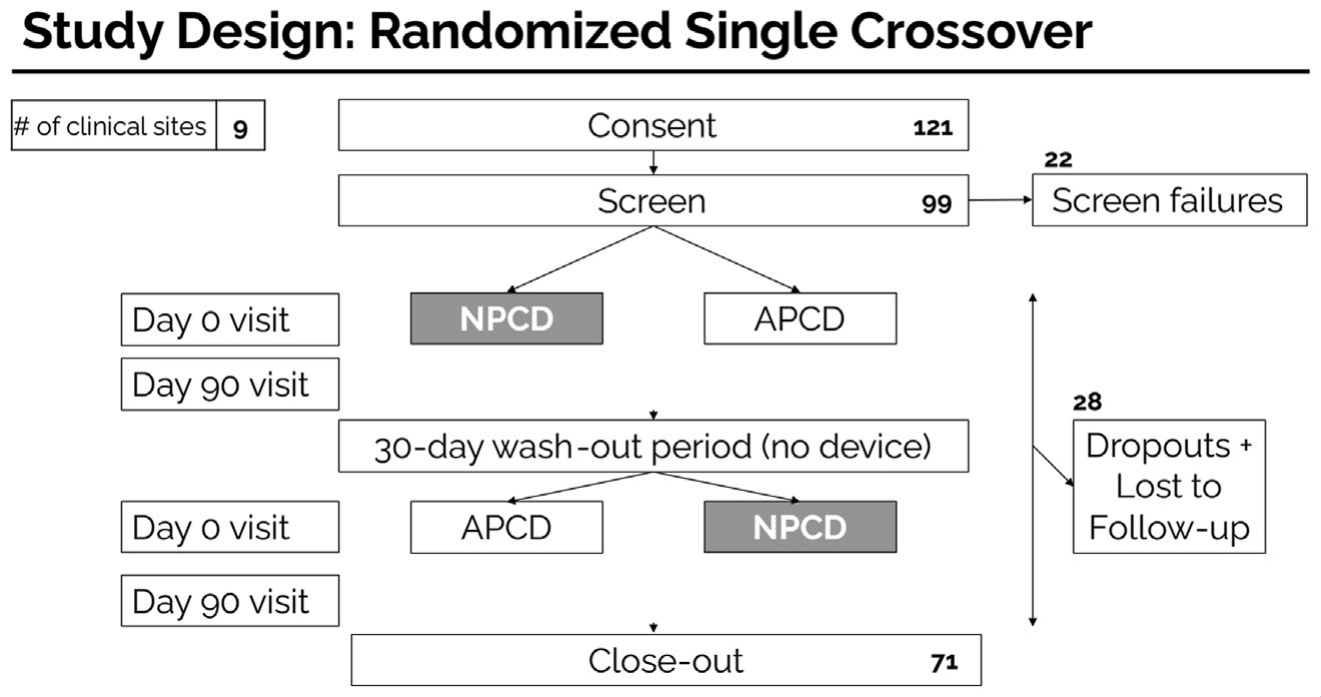
Study design. *APCD*, advanced pneumatic compression device; *NPCD*, nonpneumatic compression device.

The study design is depicted in Fig 1. An initial 30-day washout period was established in which no PCDs were used. During this period, patients were allowed to continue their conservative care, which included the use of compression garments, without any physical therapy visits. After this initial 30-day period, each patient was randomized to receive either the NPCD or the APCD treatment for 90 continuous days. At the end of the treatment duration (day 90), another 30-day washout period was established in which no PCD was used, and patients were subsequently crossed over to the alternate device treatment. For each device treatment arm, measurements were collected at day 0 and day 90, except for the patient study survey, which was performed at the end of the study. All patients were trained on how to use the devices and don/doff the respective device garments. Study devices included either the NPCD (Dayspring) or a commercially available APCD (of the 71 patients who completed the study, 2 used an Airos E0652 device, one used a Lympha press E0652 device, and the remaining 68 patients used the E0652 Flexitouch plus [PG32-G3] device). Fig 2 presents a schematic of both types of devices. Patients were instructed to use the assigned PCD once daily on the study limb for a minimum of 60 minutes. Patients were permitted to continue the use of elastic compression socks and the general duration of use was captured using the patient survey at the end of the study.

### Statistical analysis

The study was designed with the hypothesis that the NPCD would be noninferior to the APCD in decreasing volume in the affected limb in patients with lymphedema, with a noninferiority primary end point for decrease in limb volume. We used the results from previous studies^20^, ^21^, ^22^ and their measured effect sizes to estimate that a sample size of 40 patients would be adequate to demonstrate noninferiority in a randomized cross-over design.

The software packages used for data analysis for this prospective study were Microsoft Excel (Microsoft Corp, Redmond, WA) and STATA (StataCorp, College Station, TX). Changes in measured outcomes from days 0 to day 90 for both groups and categorical variables were presented as proportions, normally distributed continuous variables presented as mean ± standard error, and skewed continuous variables presented as median (interquartile range). Assumptions were checked; nonparametric alternatives were considered as needed for skewed distributions. Univariate and multivariable analyses were performed with candidate variables and outcome measures. Statistical significance was tested using a two-sided alpha level of 0.05 and with appropriate multiple testing correction (Bonferroni or Benjamini-Hochberg) approach when needed, with each limb considered a unique observation.

## Results

### Patients and demographics

A total of 121 patients were screened and 22 failed at screening; 99 patients entered the study. Over the entire study, 24 patients withdrew consent and 4 were lost to follow-up or were missing data. Of the 24 lost to follow-up, 3 dropped out of the study before assignment of a treatment device, 6 during the APCD group, and 15 during the NPCD group. In the final analysis, a total of 71 patients (108 affected limbs) were analyzed (Fig 1). The mean patient age was 58.7 ± 1.8 years, with 73% female (n = 52). The overall study population had an average body mass index of 32.6 ± 1.1. The majority of the patients (n = 60 [85%]) had secondary lower extremity lymphedema and 52.1% of patients had bilateral disease. The majority of patients presented with stage II lymphedema (stage I 18.3%, stage II 61.9%, stage III 19.7%). The mean duration of lymphedema was 8.1 ± 0.9 years. Patient demographics are summarized in Table I.

**Table I:** Patient demographics

Patients	71
Age, years	58.7 ± 1.8
Gender: Female (male)	52 (19)
Race/ethnicity	
Asian	2
Caucasian	58
African American	8
Hispanic	3
Average body mass index	32.6 ± 1.1
Primary/secondary lymphedema	11/60
Affected limbs: unilateral (left/right)/bilateral	34 (18/16)/37
Lymphedema history (years since diagnosis)	8.1 ± 0.9
Lymphedema clinical stage I, II, III	13, 44, 14
Patients with sleep apnea	34%

Values are mean ± standard error or number.

All patients had a confirmed diagnosis of lymphedema and were on conservative therapy (including, but not limited to exercise, manual lymphatic drainage, compression garments, and elevation of limb) before day 0. There were 31 patients (∼44%) who were naïve to pump or APCD treatment; the remaining 40 patients (56%) were prior users.

### Primary end points and efficacy

**Fig 3:**
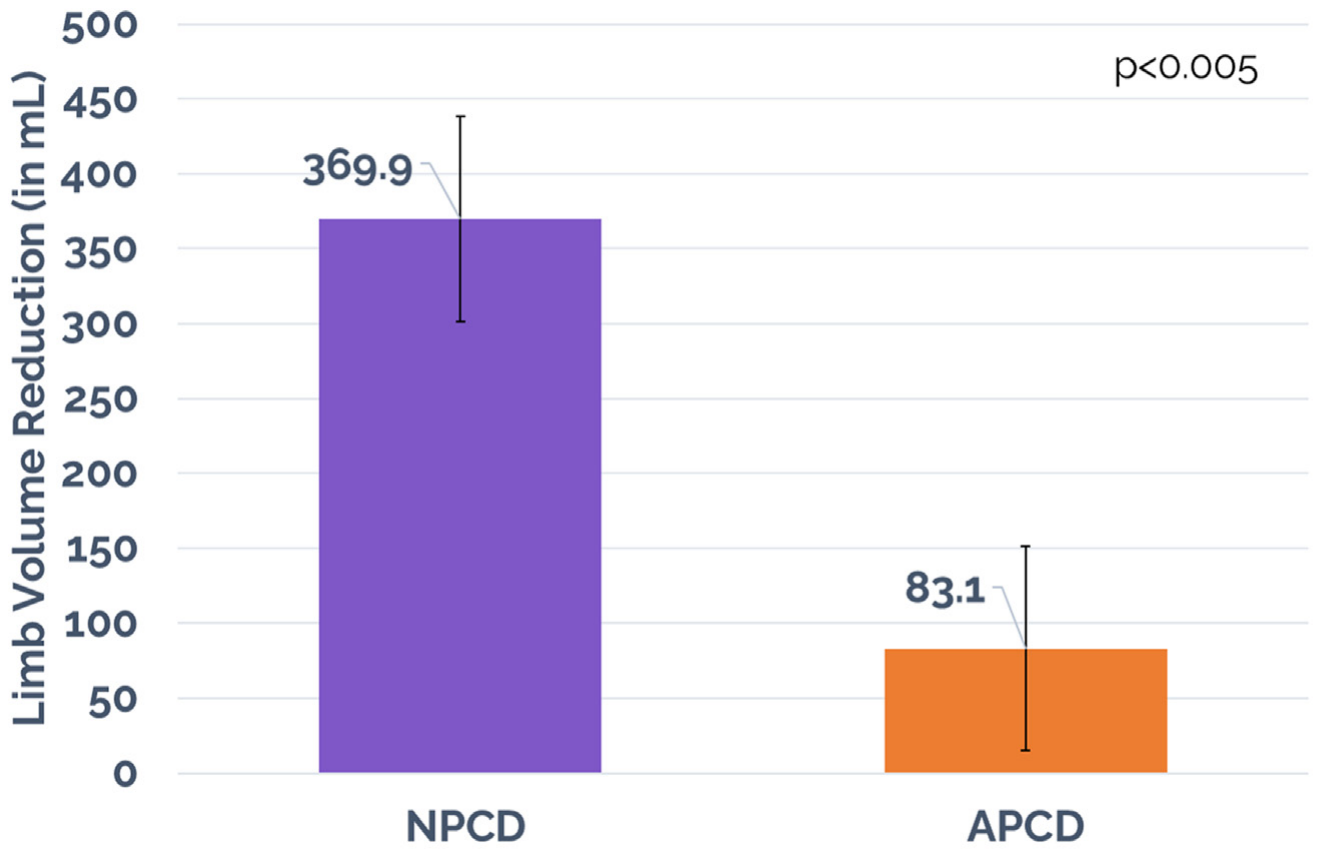
Limb volume response. *APCD*, advanced pneumatic compression device; *NPCD*, nonpneumatic compression device.

**Fig 4:**
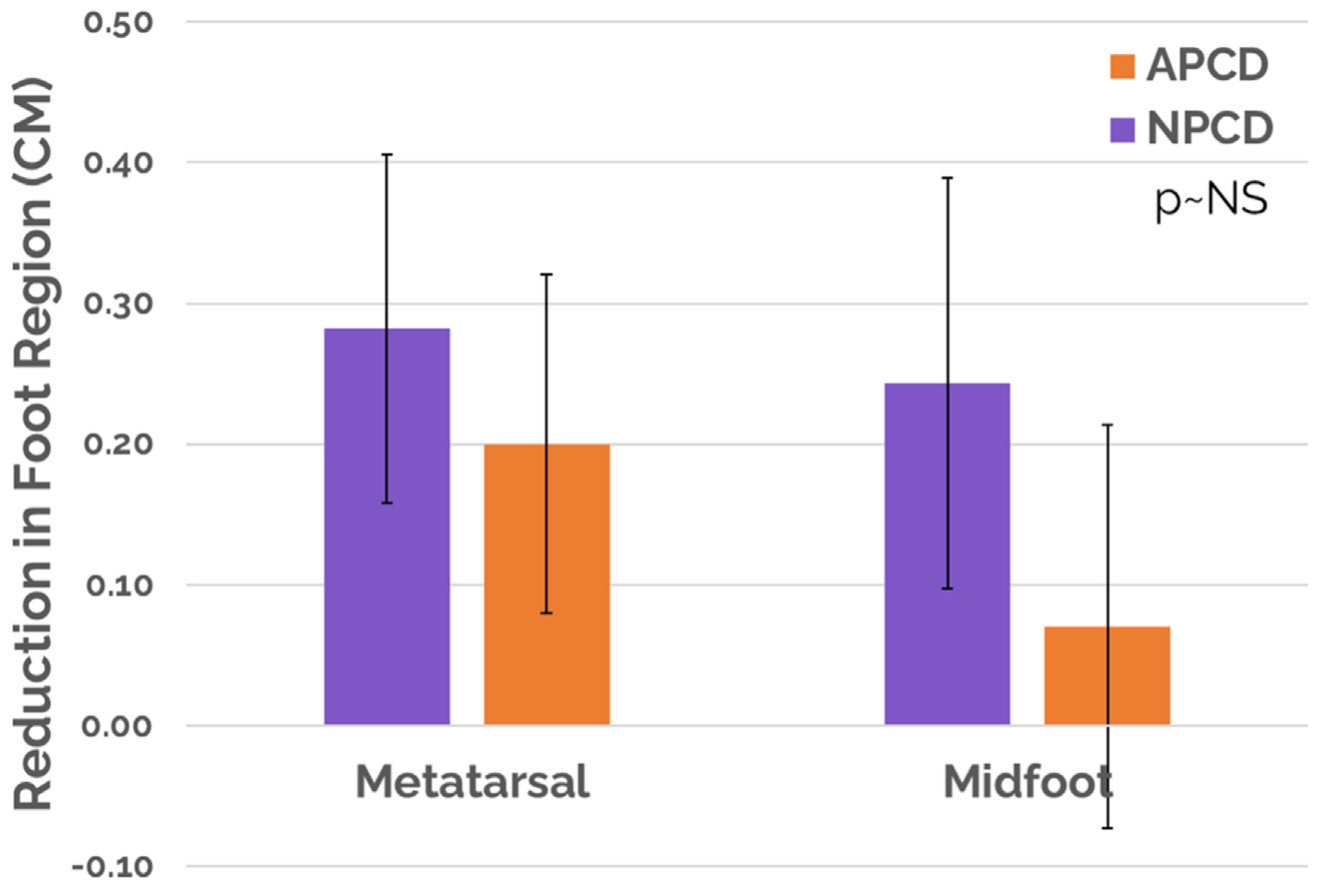
Mean change in the foot. *APCD*, advanced pneumatic compression device; *NPCD*, nonpneumatic compression device.

In the NPCD treatment arm, a mean limb volume decrease with standard error of 369.9 ± 68.19 mL (*P* < .05) and a median of 300.5 mL was achieved vs that of 83.1 ± 67.99 mL (*P* < .05) and a median of 62.0 mL for the APCD treatment arm (Fig 3). Statistical significance for comparing mean limb volume decreases between the treatment arms was achieved, favoring NPCD (*P* < .005). Changes in the foot were monitored by measurements at the metatarsal heads and midfoot for both treatment groups between day 0 and day 90, and no significant difference was detected between groups (Fig 4).

**Fig 5:**
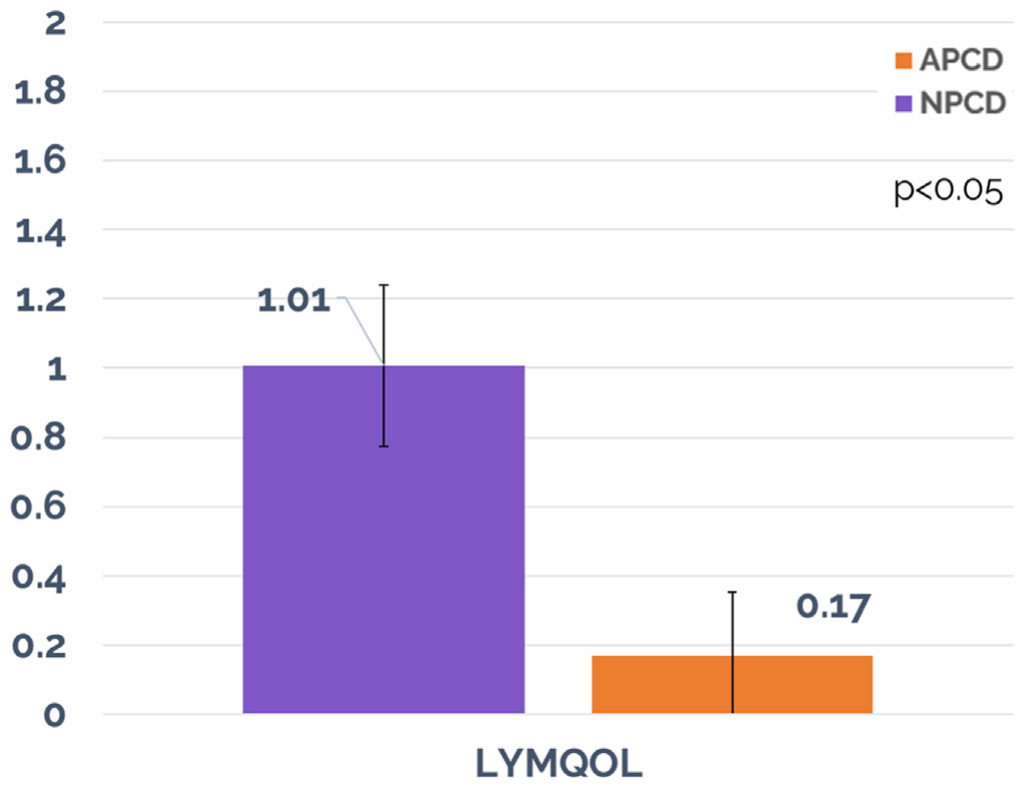
Overall Lymphedema Quality of Life Questionnaire (*LYMQOL*) score. *APCD*, advanced pneumatic compression device; *NPCD*, nonpneumatic compression device.

**Fig 6:**
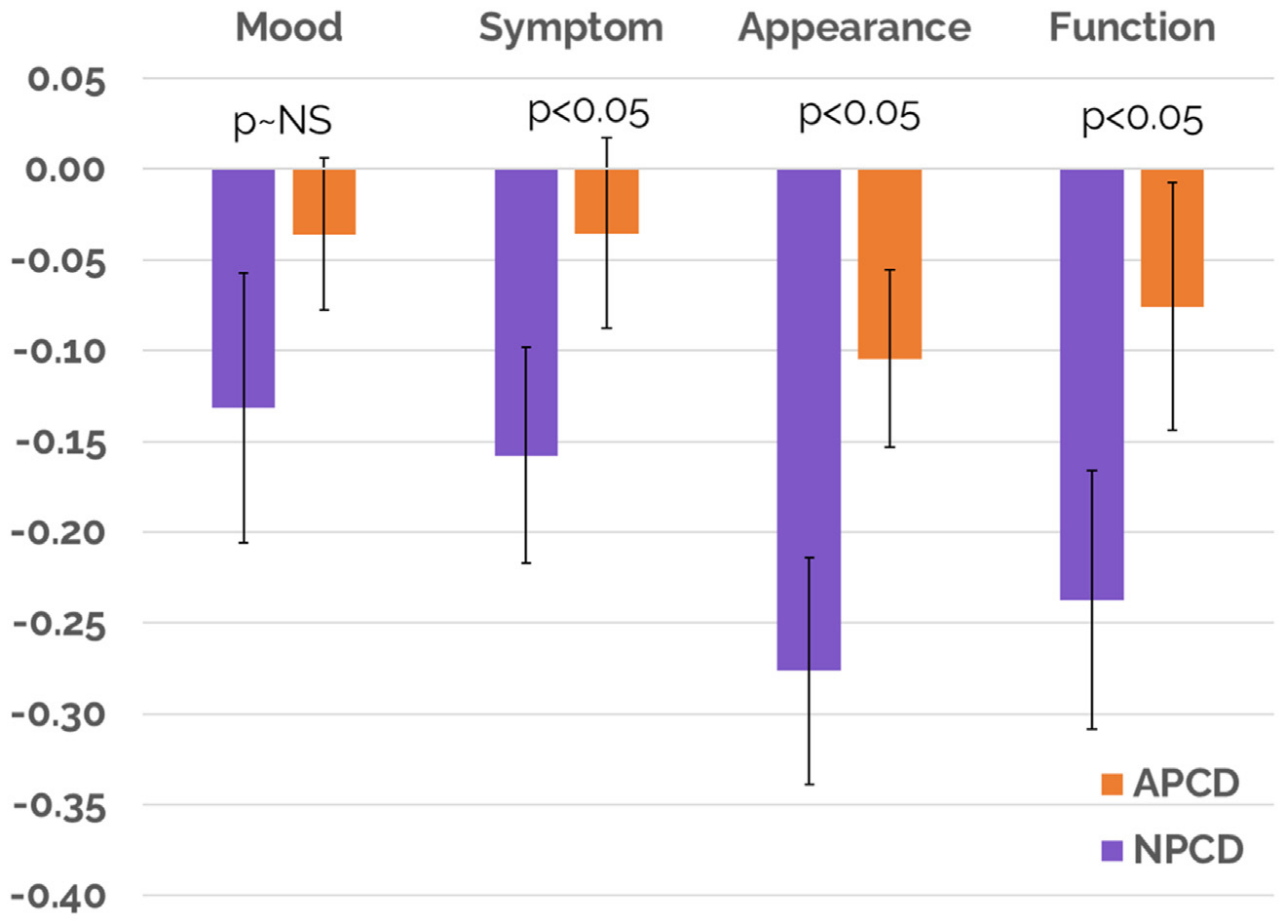
Lymphedema Quality of Life Questionnaire (*LYMQOL*) functional scores. *APCD*, advanced pneumatic compression device; *NPCD*, nonpneumatic compression device.

Significant improvement in QoL was achieved for NPCD and but not for APCD treatment. Overall LYMQOL score improvements of 1.01 ± 0.23 (*P* < .05) and a median of 1.0 for NPCD vs that of 0.17 ± 0.18 (*P* > .05) and a median of 0.0 for APCD were achieved. Statistical significance for comparing overall LYMQOL improvement between the two treatment arms was achieved, favoring NPCD (*P* < .05). Significant improvement in LYMQOL functional subscores were mixed for both treatment arms. The NPCD treatment arm achieved statistically significant improvement across all but one subscore (mood, −0.13; *P* > .05), whereas the APCD treatment arm achieved statistically significant improvement in only one subscore (appearance, −0.1; *P* < .05). Statistical significance for comparing the LYMQOL functional subscore improvements between the two treatment arms was achieved in function, appearance, and symptoms, favoring NPCD (*P* < .05), but not in mood. See Table II and Figs 5 and 6 for a summary of the primary outcomes, including LYMQOL. LYMQOL is a validated clinical tool and 1.0 point (the lowest count) in the overall score is considered clinically meaningful.

**Table II:** Volume decrease and Lymphedema Quality of Life Questionnaire (*LYMQOL*) response for all cohorts

Outcome measure	NPCD		APCD		*P* value (comparison between groups)
Reduction in limb volume, mL	369.9	<.05^a^	83.1	<.05^a^	<.05^a^
Reduction in midfoot region, cm	0.28	<.05^a^	0.20	<.05^a^	>.05
Reduction in metatarsal region, cm	0.24	<.05^a^	0.07	>.05	>.05
Overall LYMQOL	1.01	<.05^a^	0.17	>.05	<.05^a^
Function	−0.24	<.05^a^	−0.08	>.05	<.05^a^
Appearance	−0.28	<.05^a^	−0.10	<.05^a^	<.05^a^
Symptom	−0.16	<.05^a^	−0.04	>.05	<.05^a^
Mood	−0.13	>.05	−0.04	>.05	>.05
Adherence	81%		56%		<.05^a^
Reporting less compression stocking use	66%		9%		<.05^a^
Active	91%		0%		<.05^a^
Portable	97%		14%		<.05^a^
Overall preference	78%		22%		<.05^a^

*APCD,* Advanced pneumatic compression device; *LYMQOL,* Lymphedema Quality of Life Questionnaire; *NPCD,* nonpneumatic compression device.

a *P* < .05 represents statistical difference.

**Fig 7:**
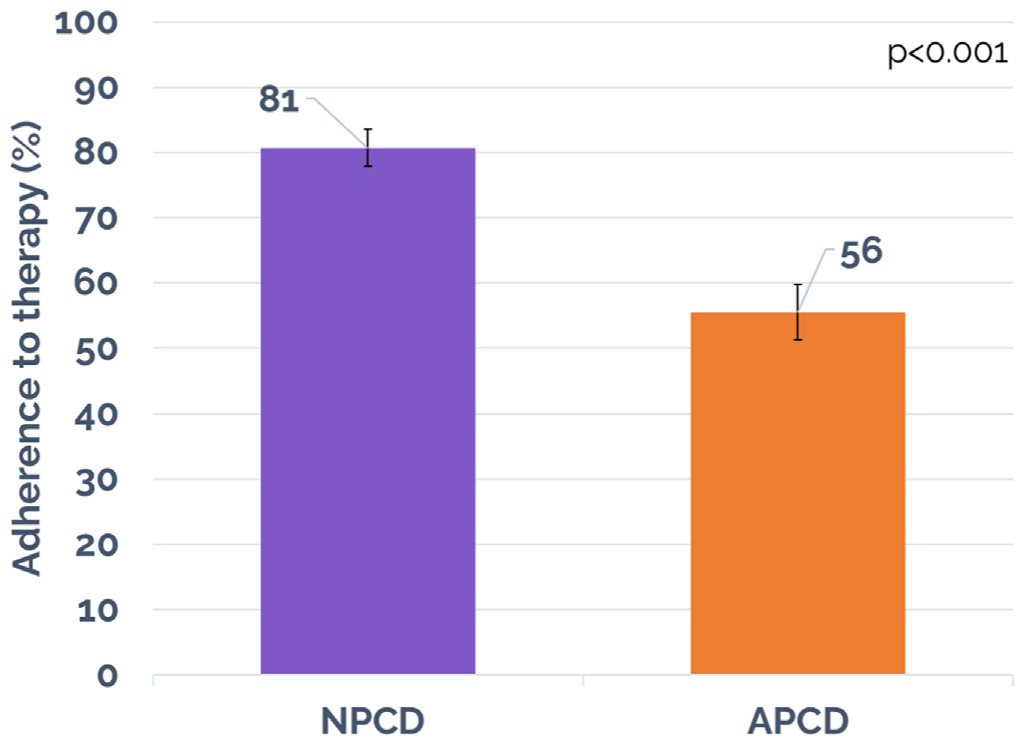
Adherence to treatment. *APCD*, advanced pneumatic compression device; *NPCD*, nonpneumatic compression device.

Treatment adherence was reported as 81.0% ± 2.9% with a median of 90% for NPCD and 56.0% ± 4.2% with a median of 60% for APCD. Statistical significance was achieved comparing adherence for the two treatment arms, favoring NPCD (*P* < .001). Fig 7 contains a graphical representation of these results. A cohort analysis was performed on a subset of patients who reported >80% adherence for both treatment arms, which included 61% of patients (43/71) in the NPCD treatment arm and 28% of patients (20/71) in the APCD treatment arm. Patients in both treatment arms in this subanalysis achieved statistically significant mean limb volume decrease (327.7 ± 83.69 mL [*P* < .05]) for NPCD and 170.1 ± 65.15 mL for APCD [*P* < .05]). In the QoL measure, both treatment arms also achieved overall LYMQOL improvement with 1.27 (*P* < .05) for NPCD and 0.63 (*P* < .05) for APCD. For the domain-specific subscores, the NPCD achieved a statistically significant improvement across all domains except for symptom, whereas the APCD achieved a statistically significant improvement only in the function domain. In secondary outcomes, similar percentages were reported for being active, device being portable as were in the overall study population, including a preference for NPCD treatment (73%) compared with 27% for APCD treatment.

Of the patients who completed the study, 49.3% (n = 35) had a diagnosis of phlebolymphedema. In this subpopulation, the mean limb volume decrease was 424.4.9 ± 100.9 mL in the NPCD cohort and 50.8 ± 112.1 mL in the APCD cohort. Improvement in overall QoL (LYMQOL) was found to be 1.39 ± 0.39 points in the NPCD cohort and 0.18 ± 0.29 in the APCD cohort. Treatment adherence was 8.01% ± 6.5% in the NPCD cohort and 49.0% ± 4.0% in the APCD cohort.

### Secondary end points and safety

No device-related AEs or device-related severe AEs were reported in either the NPCD or the APCD treatment arms. Unrelated to either devices, the following AEs were reported during the course of the study: 2 mild AEs (with a fall on ice and rolled ankle, both resolved); 12 moderate AEs (Mohs surgery, torn calf muscle, allergy to medication, COVID, sciatic leg pain, ankle sprain, implantation of a heart loop recorder, knee injections for pain, cellulitis, allergy to Bactrim; all resolved with medical or surgical intervention; and cancer recurrence managed with ongoing medical intervention); 15 moderate severe AEs (cardiac arrythmia, hospitalization, pacemaker implantation, neck pain/fusion with rod placement, fall with metatarsal break and numbness, torn meniscus, urinary tract infection requiring hospitalization, urinary retention, retinal surgery, cataract surgery, wound vac treatment, and stent placement; all resolved with medical or surgical intervention).

No truncal swelling or worsening was reported (compared with baseline) for any patients for either group.

**Fig 8:**
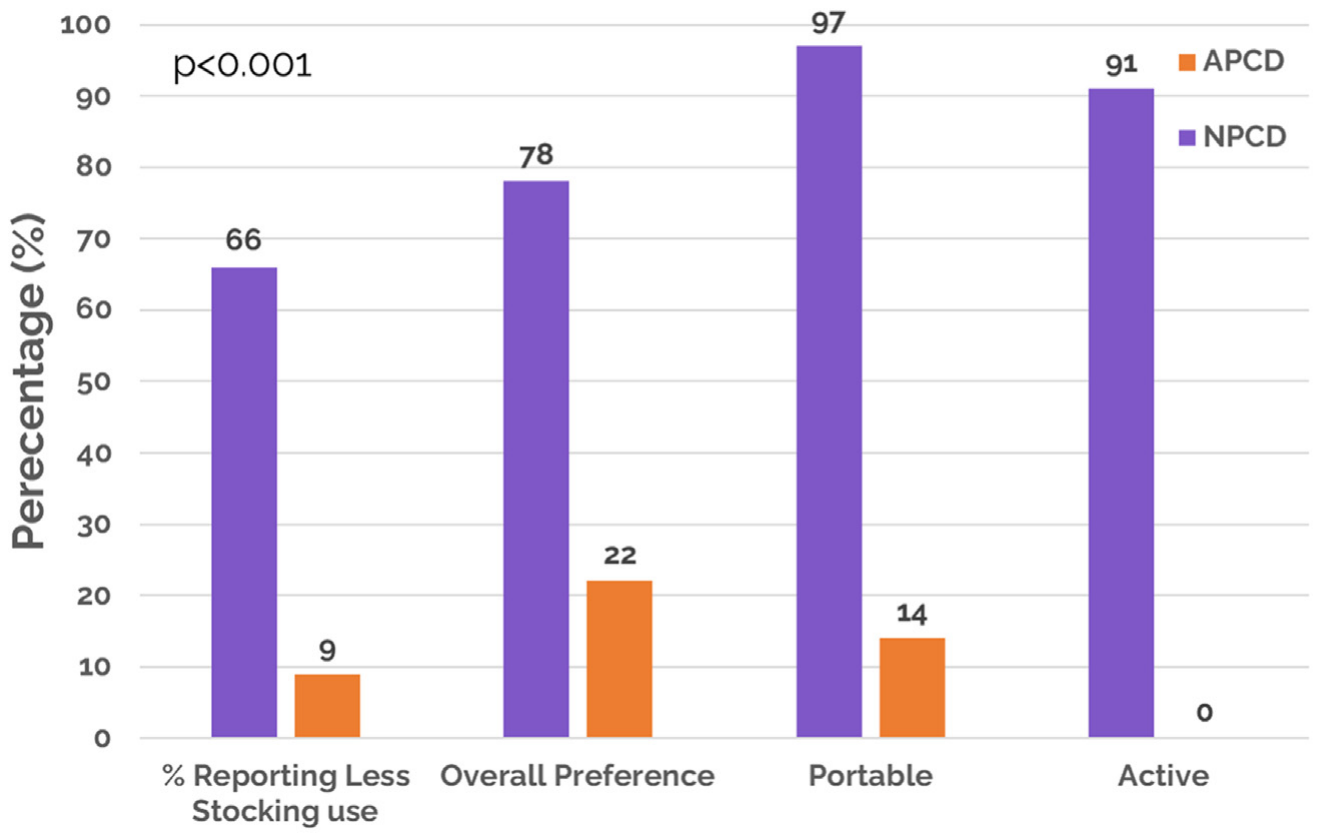
Patient preference questionnaire results. *APCD*, advanced pneumatic compression device; *NPCD*, nonpneumatic compression device.

For the patient survey, which was administered at the end of the study, a majority of the patients (91%) reported being active during NPCD treatment (0% for APCD treatment) and 78% of patients responded preferring NPCD as their treatment choice compared with 22% who preferred APCD treatment. In addition, 66% of patients on NPCD treatment reported decreased use of compression stockings compared with 9% of patients on APCD treatment reporting decreased use of compression stockings (Fig 8).

### Disease-related health episodes and resource use

Select disease-related health episode and resource use data were also collected at the beginning of the study and captured after 90 days of treatment with each device. Baseline average number of episodes in the 12 months before study enrollment was 0.6 ± 0.1 for cellulitis and 0.3 ± 0.1 for ulceration. For resource use, the baseline average number of days in the 12 months before study enrollment for hospitalization associated with complications from lymphedema was 1.0 ± 0.4, and for use of compression stockings, it was 304.3 ± 14.6. The average number of lymphedema-related physical therapy visits in the 12 months before study was 19.5 ± 3.7.

During the NPCD treatment period, no episodes of cellulitis, ulceration, or hospitalization were reported. Average lymphedema-related physical therapy visits during this 90-day study period were found to be approximately 0.2 visits for the NPCD group.

During the APCD treatment period, there were a total of three cases (∼4%) of cellulitis reported and one case of ulceration reported (∼1%), all of which were resolved with medical intervention. A total of 8 hospitalization days were also reported during APCD treatment period. The average number of lymphedema-related physical therapy visits during this 90-day study period was found to be approximately 2.6 visits in the APCD group.

Additionally, in the subset of treatment-naïve (have never used a PCD in their past) population of (31 patients) in the NPCD treatment arm, a mean limb volume decrease with standard error of 266.4 ± 92.02 mL (*P* < .05) was achieved vs that of 162.5 ± 76.14 mL (*P* < .05) for the APCD treatment arm. The overall LYMQOL score improvements of 1.15 ± 0.46 (*P* < .05) for NPCD vs that of 0.50 ± 0.34 (*P* > .05) for APCD were achieved. Treatment adherence was reported as 80.0% ± 4.3% for NPCD and 55.0% ± 6.7% for APCD, which follows the general trend observed in the greater patient population of the study.

## Discussion

In this randomized multicenter crossover trial, comparative treatment effectiveness between a novel NPCD (Dayspring, Koya Medical) and a APCD (Flexitouch Plus, Tactile Medical) for the treatment of lower extremity lymphedema was investigated. Patients with NPCD treatment found a significantly greater decrease in limb volume (369.9 mL) compared with limb volume decrease with the APCD treatment (83.1 mL). Statistical significance for comparing the mean limb volume decreases between the treatment arms was achieved, favoring a superior outcome for NPCD (*P* < .005). Additionally, significant improvements in overall QoL were only achieved in the NPCD treatment arm.

Our study was representative of typical patients with lower extremity lymphedema. The distributions of secondary vs primary lymphedema, the mean age, unilateral vs bilateral, as well as lymphedema staging seem to be similar in our study compared with a review of patients with lower extremity lymphedema conducted by Dean et al,^3^ although the body mass index of patients in our study (32.6 ± 1.1) was less than that of the previous study (40.2 ± 14.8).

In selecting treatment options for chronic conditions such as lymphedema, for which there is no cure, considerations for the treatment's impact on patient's QoL cannot be understated. In this trial, significant clinical advantages were seen in the NPCD treatment arm in terms of overall performance, including in mean limb volume decrease, as well as improvements in QoL and adherence. It is possible that NPCD's superior QoL and adherence outcomes are due to the compact nature and portability of the device and its mobile power source allowing the patient to be and remain mobile so they may address their basic daily living activities, thus providing more treatment opportunities resulting in a favorable cycle that is then repeated. In contrast, APCDs are typically not as portable, much bulkier, and the treatment's immobilization requirement owing to the need to be affixed to a stationary power source may all play a role in deterring its use.

In terms of the superior outcome in mean limb volume decrease observed in the NPCD treatment arm, it is possible that the differentiated multimodal mechanisms provided by NPCD, which includes static compression, gradient sequential compression, and the allowance for muscle and joint contractions, especially in the calf muscle for lower extremity lymphedema patients, may together provide a meaningful and relevant clinical amplification effect previously unobserved. The calf muscle pump is responsible for the majority of venous return to the central venous system,^24^ with both a high capacitance and an ejection fraction of 65%. Patients with venous reflux develop symptoms because the rate of venous flow increases by as much as five-fold owing to retrograde flow, consequently exceeding the output of the calf muscle pump. Exercise increases the ejection fraction and thus overall venous return throughout both the leg and foot in patients with venous insufficiency.^25^^,^^26^ This beneficial effect of exercise on the calf muscle pump system has been demonstrated in venous stasis as well as lymphedema, likely owing to the interrelated nature of the venous and lymphatic systems and the effect of tissue edema and distension on both skin fibrosis and pain.^27^ Although the combination of these different management approaches (static compression through compression garments, sequential gradient compression, and prescribed exercise) are often cited and recommended in the various clinical guidelines,^28^ no single compression treatment option until NPCD has provided the means or opportunities for which they can be accomplished simultaneously.

These hypotheses seem to support the results from the subanalysis reported elsewhere in this article, in which adherence was controlled for both treatment arms. When looking only at those patients who reported using the respective devices ≥80% of the time, although underpowered, the superior outcomes in NPCD treatment arm was sustained in the primary outcome measures.

Finally, and perhaps most important, the impact of adherence on outcome is critically relevant in today's cost-conscious health care system, particularly when considering that the costs between NPCD and APCD are comparable. If a patient is not going to use a device, its efficacy in a controlled environment, even a randomized clinical trial, is moot and only adds financial strain to patient and to health economics as a whole. Indeed, a home health device is only as good as its adoption in the patient's daily life.

### Limitations

**Fig 9:**
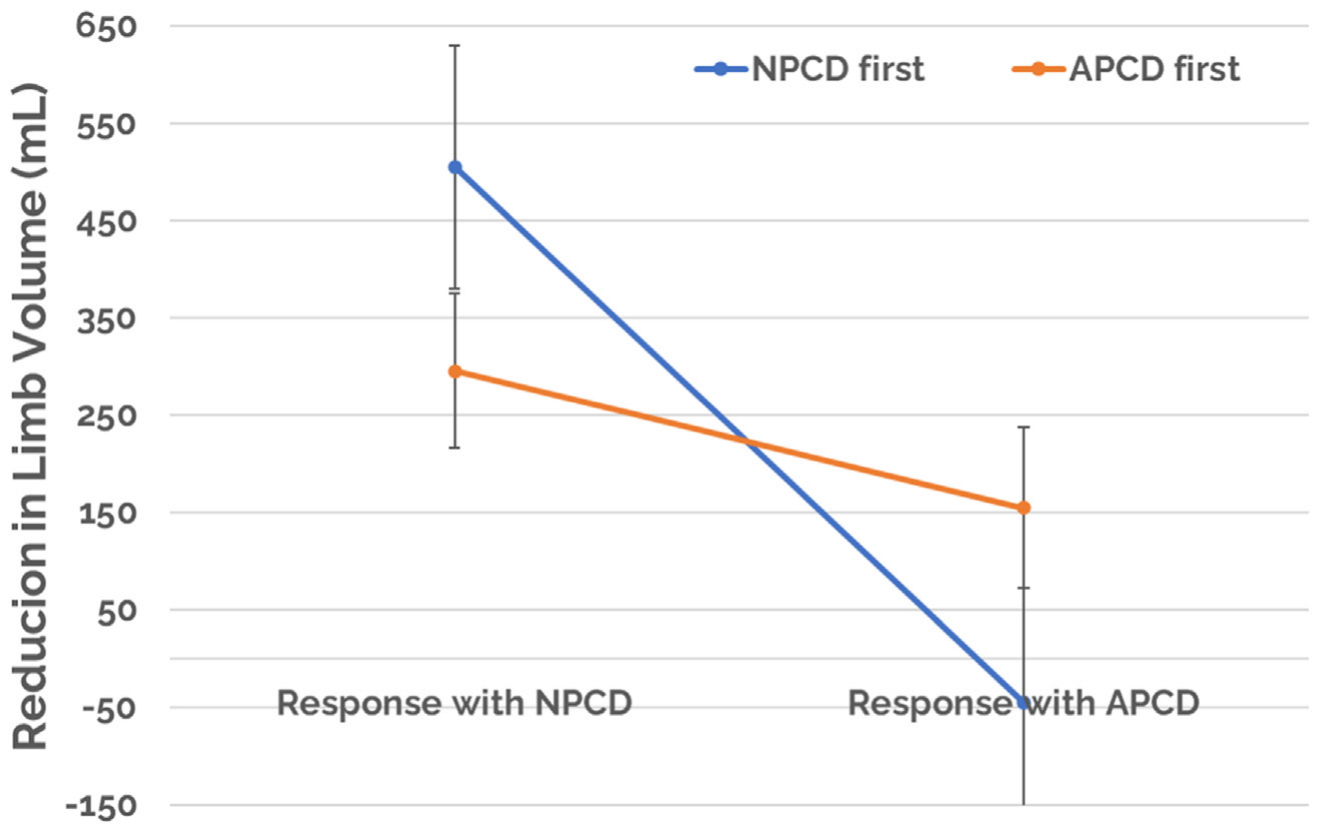
Limb volume decrease as a function of first device used. *APCD*, advanced pneumatic compression device; *NPCD*, nonpneumatic compression device.

**Fig 10:**
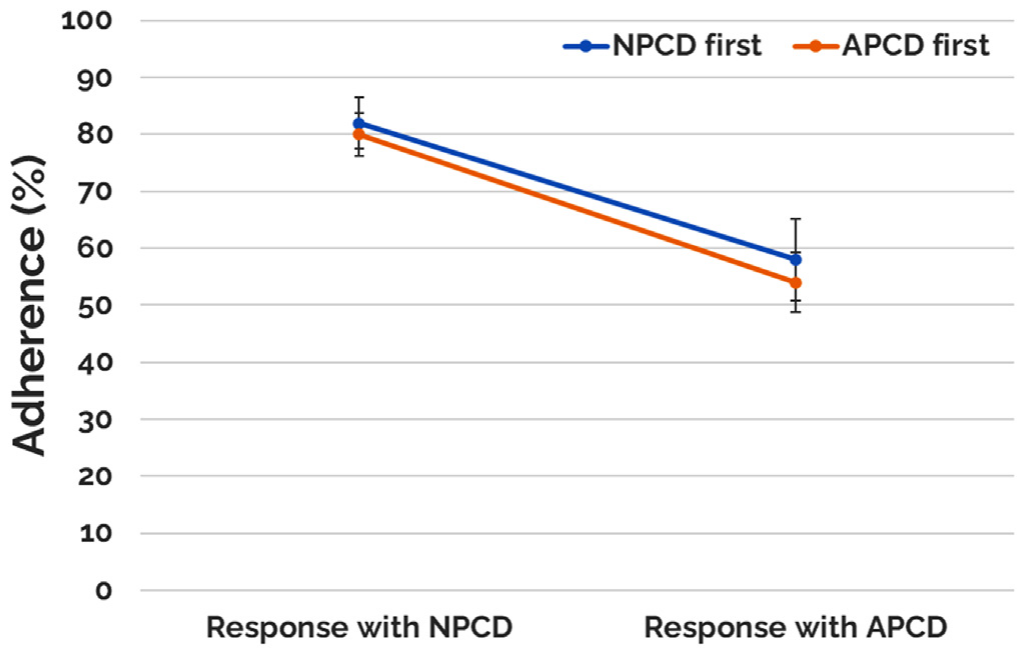
Adherence as a function of first device used. *APCD*, advanced pneumatic compression device; *NPCD*, nonpneumatic compression device.

**Fig 11:**
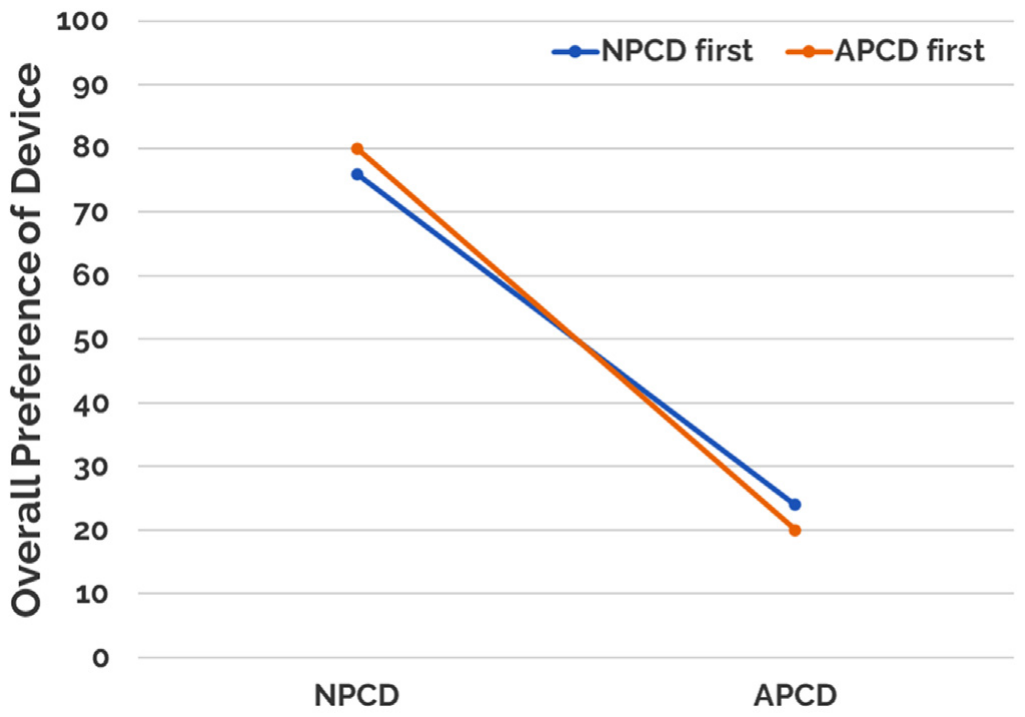
Overall preference as a function of first device used. *APCD*, advanced pneumatic compression device; *NPCD*, nonpneumatic compression device.

Common biases in crossover studies include unintended biases such as order of which device was first used and whether there is an unforeseen carryover effect from one treatment arm to the comparator. Additional analyses were performed to examine adherence and mean limb volume decrease with respect to whether the patient was treated with NPCD or with APCD. For treatment adherence, there seems to be negligible differences with regard to which device treatment the patient started on first. Regardless of the order of device used, patients reported treatment adherence between 80% and 82% while on NPCD and 54% to 58% while on APCD (Fig 9). For mean limb volume, decreases achieved with NPCD ranges from 296.1 mL (*P* < .05) to 505.8 mL (*P* < .05) and (44.1 mL volume increase) (*P* > .05) to 155.2 mL (*P* < .05) for APCD (Fig 10). For treatment preference, there seems to be negligible differences with regard to which device treatment, the patient started on first (Fig 11). Additionally, despite the initial 30-day washout period, some patients who have prior experience with an APCD may retain preconceived notions about that device, which may have influenced compliance.

Although there are no randomized controlled studies for APCDs, Maldonado et al^29^ have shown that APCDs achieved a meaningful decrease in limb volume decrease at 12 weeks. However, the impact on QoL from APCDs was not detected until week 52.^29^ In this trial, the time to impact of APCD on mean limb volume decrease as well as the lack of impact on QoL seem to corroborate those in earlier findings. Furthermore, it is possible that the underlying mechanistic differences between the two treatment modalities (NPCD vs APCD) allow practitioners to see improvements earlier with the former, which is encouraging. Finally, the additional health resource use and episodic data collected at baseline seem to favor the NPCD treatment arm, but are not statistically powered sufficiently to detect significant changes.

## Conclusions

Lower extremity lymphedema often develops in the setting of a damaged or a poorly functioning venous system. Although compression and elevation are often the mainstays of treatment recommendations, there is a treatment gap for lymphedema that not only considers the objective clinical outcomes such as limb volume, but also the QoL and adherence choices that patients make to perform the treatment each day.

In this randomized comparative treatment effectiveness study (TEAYS), patients in the NPCD (Dayspring, Koya Medical) treatment arm achieved superior outcomes in both mean limb volume decrease as well as in QoL when compared with when they were on the APCD (Flexitouch Plus) treatment arm. The superior outcomes associated NPCD are similar to those previously published for the upper extremities^22^ and may be a result of underlying treatment mechanistic differences and, as importantly, the improved ability of the patient to adhere to the NPCD treatment. The marked improvements in outcomes as well as the time to which these improvements were observed with the NPCD treatment in this study, taken together with prior studies on NPCD, represent a differentiated and superior treatment choice for managing patients with lower extremity lymphedema.

## Author Contributions

Conception and design: MB, RW, TB, SR, TM

Analysis and interpretation: SR, TM

Data collection: MB, RW, TB, SD, VR, NC, SR, TM

Writing the article: TM

Critical revision of the article: MB, RW, TB, SD, VR, NC, SR, TM

Final approval of the article: MB, RW, TB, SD, VR, NC, SR, TM

Statistical analysis: SR, TM

Obtained funding: Not applicable

Overall responsibility: TM

SR and TM contributed equally to this article and share senior authorship.

## Funding

This work was funded by Koya Medical. Koya medical was not involved in manuscript writing. Koya Medical was not involved in the decision to submit the manuscript for publication.

## Disclosures

S.G.R. and T.S.M. serve on advisory board and receive consulting fees and currently own no shares of Koya Medical, manufacturer of Dayspring.
